# In Vitro Antimicrobial Studies of Mesoporous Silica Nanoparticles Comprising Anionic Ciprofloxacin Ionic Liquids and Organic Salts

**DOI:** 10.3390/pharmaceutics15071934

**Published:** 2023-07-12

**Authors:** Luís Filipe, Telma de Sousa, Dário Silva, Miguel M. Santos, Manuela Ribeiro Carrott, Patrícia Poeta, Luís C. Branco, Sandra Gago

**Affiliations:** 1 Associated Laboratory for Green Chemistry (LAQV) of the Network of Chemistry and Technology (REQUIMTE), NOVA School of Science and Technology (FCT NOVA), Campus da Caparica, 2829-516 Caparica, Portugal; lm.filipe@campus.fct.unl.pt (L.F.); telmas@utad.pt (T.d.S.); dmv.silva@campus.fct.unl.pt (D.S.); miguelmsantos@fct.unl.pt (M.M.S.); ppoeta@utad.pt (P.P.); 2 Department of Genetics and Biotechnology, University of Trás-os-Montes and Alto Douro (UTAD), 5000-801 Vila Real, Portugal; 3 Microbiology and Antibiotic Resistance Team (MicroART), Departamento de Ciências Veterinárias, Universidade de Trás-os-Montes e Alto Douro, 5000-801 Vila Real, Portugal; 4 Functional Genomics and Proteomics Unit, University of Trás-os-Montes and Alto Douro (UTAD), 5000-801 Vila Real, Portugal; 5 LAQV-REQUIMTE, Institute for Research and Advanced Studies, Department of Chemistry and Biochemistry, School of Sciences and Technology, University of Évora, 7000-671 Évora, Portugal; manrc@uevora.pt; 6 Veterinary and Animal Research Centre (CECAV), University of Trás-os-Montes and Alto Douro (UTAD), 5000-801 Vila Real, Portugal; 7 Associate Laboratory for Animal and Veterinary Sciences (AL4AnimalS), University of Trás-os-Montes and Alto Douro (UTAD), 5000-801 Vila Real, Portugal

**Keywords:** ciprofloxacin, ionic liquids, mesoporous silica nanoparticles, toxicity, antimicrobial activity, antibiotic resistance

## Abstract

The combination of active pharmaceutical ingredients in the form of ionic liquids or organic salts (API-OSILs) with mesoporous silica nanoparticles (MSNs) as drug carriers can provide a useful tool in enhancing the capabilities of current antibiotics, especially against resistant strains of bacteria. In this publication, the preparation of a set of three nanomaterials based on the modification of a MSN surface with cholinium ([MSN-Chol][Cip]), 1-methylimidazolium ([MSN-1-MiM][Cip]) and 3-picolinium ([MSN-3-Pic][Cip]) ionic liquids coupled with anionic ciprofloxacin have been reported. All ionic liquids and functionalized nanomaterials were prepared through sustainable protocols, using microwave-assisted heating as an alternative to conventional methods. All materials were characterized through FTIR, solution ^1^H NMR, elemental analysis, XRD and N_2_ adsorption at 77 K. The prepared materials showed no in vitro cytotoxicity in fibroblasts viability assays. The minimum inhibitory concentration (MIC) for all materials was tested against Gram-negative *K. pneumoniae* and Gram-positive *Enterococcus* spp., both with resistant and sensitive strains. All sets of nanomaterials containing the anionic antibiotic outperformed free ciprofloxacin against resistant and sensitive forms of *K. pneumoniae*, with the prominent case of [MSN-Chol][Cip] suggesting a tenfold decrease in the MIC against sensitive strains. Against resistant *K. pneumoniae*, a five-fold decrease in the MIC was observed for all sets of nanomaterials compared with neutral ciprofloxacin. Against *Enterococcus* spp., only [MSN-1-MiM][Cip] was able to demonstrate a slight improvement over the free antibiotic.

## 1. Introduction

Throughout recent decades, an exponential increase in multidrug resistant bacterial infections has been observed, which has hampered the efficacy and proficiency of current antibiotics and therapies. According to a Centers for Disease Control and Prevention (CDC) report [1], antibiotic resistance is now claiming up to 700,000 human lives every year, with the situation being particularly dire in developing countries. If no actions are taken to tackle this emerging problem, the World Health Organization (WHO) warns that antibiotic resistance might start reaching death tolls on the order of 10 million people yearly [2].

While there are many different reasons for the failure of current antibiotics, irresponsible use in sectors such as livestock is recognized to be promoting the natural selection of increasingly resistant bacterial strains. Furthermore, current antibiotics fail to reach the desired concentrations at the site of infection due to low permeability or bioavailability, which are also known limitations of current therapies [3]. In recent years, a new class of active pharmaceutical ingredients (APIs) combined with biocompatible ionic systems, such as organic salts and ionic liquids (OSILs), has been increasingly reported in the literature with very promising results. This new class of compounds—API-OSILs—holds the possibility to improve a pharmaceutical drug’s most limiting properties, such as crystallinity (polymorphism), solubility, permeability and bioavailability, while maintaining or even increasing the desired antimicrobial effect [4,5]. Our research team already reported the potential of preparing and characterizing several API-OSILs formulations ranging from β-lactams [6,7,8,9,10], fluoroquinolones [10,11,12,13], non-steroid anti-inflammatory drugs (e.g., ibuprofen [10,14] or naproxen [10]) and even bone antiresorptive agents such as zoledronic [15] and alendronic acids [16]. Of particular interest to the present article are the recent efforts from our research group, which have focused on the combination of fluoroquinolones such as ciprofloxacin with several biocompatible organic counter-ions. In previous work, ciprofloxacin was combined in the cationic form with methanesulfonate, gluconate and glycolate anions [11,13], and the anionic form was combined with cations such as pyridinium, ammonium [12] or *N*-alkylimidazolium derivatives [12,13] to achieve formulations with improved physical-chemical properties (e.g., increased solubility, permeability and loss of polymorphism). Furthermore, these recent publications have demonstrated the enormous versatility of API-OSILs and the possibility to tune and achieve enhanced tailor-made drugs through the conjugation of different cation–anion pairs.

The incorporation of pharmaceutical drugs in nanostructured delivery systems, such as mesoporous silica nanoparticles (MSNs), has also been increasingly reported in the literature as a very prominent area of research for drug delivery [17,18,19,20,21]. It is well established that nanoparticles can act as stable, tunable and highly-efficient drug carriers, owing to their high specific surface area and volume, tunable surface charge and pore size, high loading capacity, good biocompatibility, high chemical stability and the easy functionalization of inner pore and surface [22]. Furthermore, the use of nanoparticles to treat bacterial infections, namely resistant bacteria, has been consistently gathering attention in the literature, as very promising alternatives or complements to enhance the activity of antibacterial drugs are being reported [23,24,25]. In addition, the combination of MSNs with levofloxacin [26,27,28,29,30,31], gentamicin [32,33,34], isoniazid [35,36], rifampicin [37,38,39,40], vancomycin [41], polymyxin B [42], amoxicillin [43], cefepime and meropenem [44], moxifloxacin [45,46,47], ampicillin [48], kanamycin [49], tetracycline [50,51,52], ciprofloxacin [53,54,55,56,57] or even the synergistic combination of multiple antibiotics simultaneously [58] have been reported. In all these approaches, a consistent and reliable improvement in the efficacy of the antibiotics has been noted, granting nanoparticles a very promising future in the pharmaceutical industry. Of particular interest to the current work are the examples of combination of MSNs with ciprofloxacin, which have been developed through surface functionalization with sulfonate [53,59], silane [60,61], lipids [57], amino acids [56] or polymers [62,63]. On a fundamental note, the different sizes of the pores and the volumes have also been reported in the literature as key factors for optimal ciprofloxacin loading and release [64,65].

Herein, a complementary study using a series of non-toxic antimicrobial materials obtained through covalent modifications of the surface of MSNs with API-OSILs pairs [66] is presented. While we have noted that this method has been partially replicated elsewhere [61] with good antimicrobial results, to the best of our knowledge, the combination of MSNs and API-OSILs, especially against resistant strains of bacteria, remains a highly unexplored subject in the overall literature.

## 2. Materials and Methods

### 2.1. Materials and Methods

Tetraethyl orthosilicate (TEOS, Aldrich, 98%), hexadecyltrimethylammonium bromide (CTAB, BDH Chemicals, Radnor, PA, USA), Pluronic F127 (Aldrich, Munich, Germany), triethanolamine (TEA, Alfa Aesar, Ward Hill, MA, USA), 2-dimethylaminoethanol (Aldrich, 99.5%), 1-methylimidazole (Alfa Aesar, 99%), 3-picoline (Carlo Erba, 98%), (3-chloropropyl)triethoxysilane (TCI, 97%), ciprofloxacin (Cipro, Fluka, 98%) and NaOH were used. Microwave irradiation was performed using an Antón-Parr Monowave 450 oven with potency control. FT-IR spectra were recorded on a Bruker Tensor 27 Spectrometer in the 400–4000 cm^−1^ region, using KBr pellets. Solution ^1^H NMR spectra were obtained with a Bruker AMX400 at 400.13 MHz. Elemental analyses (EA) were carried out with a Thermofinnigan Flash EA 112 series. Nitrogen adsorption–desorption isotherms were determined at 77 K on a Quantachrome Quadrasorb equipped with a turbomolecular pump, using helium 4.6 and nitrogen 5.0 supplied by Linde Portugal. Prior to the adsorption measurements, the samples were outgassed for 8 h at 393 K achieved using a heating rate of 1 K min^−1^. X-ray diffraction (XRD) patterns were obtained using a Bruker AXS-D8 Advance diffractometer, with CuKα radiation (40 kV and 30 mA), step size of 0.01° (2θ) and 5 s per step.

### 2.2. Description of the Synthesis

Pristine mesoporous silica nanoparticles (MSNs-OH) were prepared following the procedure described by Bouchoucha et al. [67] with the minor modifications reported previously [66]. Triethoxysilane Cholinium, 1-Methylimidazolium and 3-Picolinium-based ionic liquid derivatives were prepared by microwave irradiation, as described below. MSNs-OH were then functionalized with these ionic liquids, also using microwave-assisted procedures.

#### 2.2.1. Synthesis of Triethoxysilane Cholinium Derivative (Si-[Chol][Cl])

3-(chloropropyl)triethoxysilane (8 mL, 33.3 mmol, 1.1 equivalents) and 2-dimethylaminoethanol (3 mL, 30.0 mmol) were added to a wide-neck glass vial. The reaction mixture was heated under microwave irradiation for 30 min at 85 °C until the formation of a single-phased yellow and viscous oil. The crude was then washed with diethyl ether and dried under vacuum to give 9.15 g (85%) of the pure final product designated as Si-[Chol][Cl].

**Figure 1:**
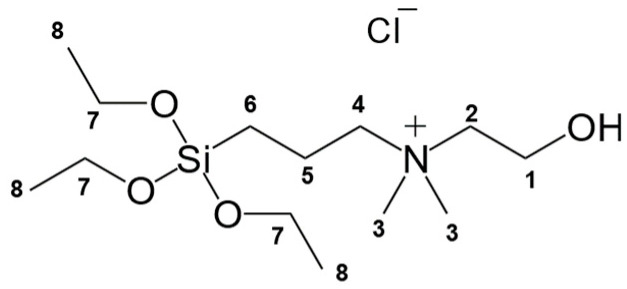
Proposed numbering scheme for triethoxysilane cholinium ionic liquid Si-[Chol][Cl].

^1^H NMR (400 MHz, CDCl_3_, rt) δ = 4.08 (s, 2H, H-1), 3.90–3.80 (m, 6H, H-3), 3.66 (q, 4H, H-2, H-4), 3.44–3.35 (m, 6H, H-7), 1.91–1.81 (m, 2H, H-5), 1.27–1.18 (m, 9H, H-8), 0.66 (t, 1H, H-6) ppm (see Figure 1 for proposed numbering).

#### 2.2.2. Synthesis of Triethoxysilane 1-Methylimidazolium Derivative (Si-[1-MiM][Cl])

3-(chloropropyl)triethoxysilane (7.54 mL, 41.3 mmol, 1.1 equivalents) and 1-methylimidazole (3 mL, 3.09 g, 37.6 mmol) were added to a wide-neck glass vial. The reaction mixture was heated under microwave irradiation for 60 min at 145 °C until the formation of a single-phased yellow and viscous oil. The crude was then washed with diethyl ether and dried under vacuum to give 10.05 g (95%) of the pure final product designated as Si-[1-MiM][Cl].

**Figure 2:**
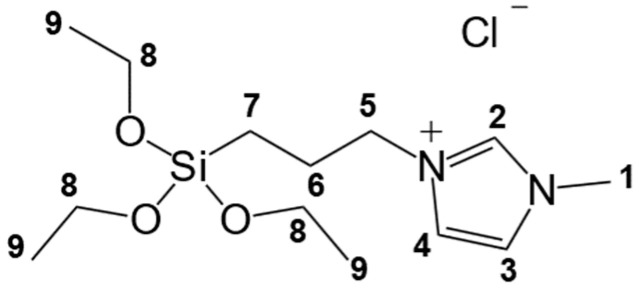
Proposed numbering scheme for triethoxysilane 1-methylimidazolium ionic liquid Si-[1-MiM][Cl].

^1^H NMR (400 MHz, CDCl_3_, rt) δ = 10.53 (s, 1H, H-2), 7.43–6.94 (dd, 2H, H-3, H-4), 4.35 (t, 2H, H-5), 4.10 (s, 2H, H-6), 3.85 (q, J = 7.0 Hz, 6H, H-8), 3.70 (s, 3H, H-1), 1.24 (t, J = 7.0 Hz, 9H, H-9), 0.70–0.61 (m, 2H, H-7) ppm (see Figure 2 for proposed numbering).

#### 2.2.3. Synthesis of Triethoxysilane 3-Picolinium Derivative (Si-[3-Pic][Cl])

3-(chloropropyl)triethoxysilane (8 mL, 33.3 mmol, 1.08 equivalents) and 3-picoline (3 mL, 30.8 mmol) were added to a wide-neck glass vial. The reaction mixture was heated under microwave irradiation for 60 min at 120 °C until the formation of a single-phased dark orange and viscous oil. The crude was then washed with diethyl ether and dried under vacuum to give 9.68 g (91%) of the pure final product designated as Si-[3-Pic][Cl].

**Figure 3:**
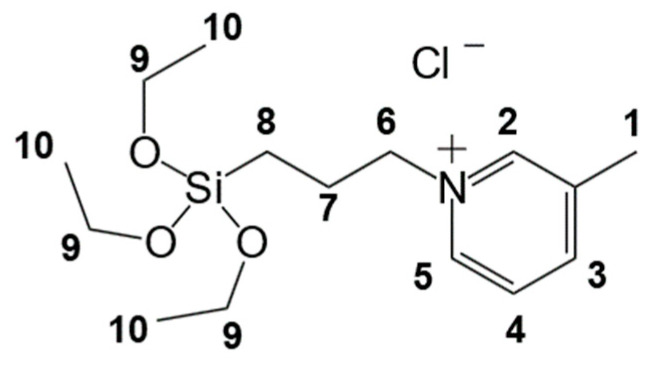
Proposed numbering assignment on the triethoxysilane 3-picolinium ionic liquid Si-[3-pic][Cl].

^1^H NMR (400 MHz, CDCl_3_, rt) δ = 9.29 (s, 1H, H-2), 9.18 (d, 1H, H-5), 8.25 (d, 1H, H-3), 8.01 (t, 1H, H-4), 4.84 (t, J = 7.2 Hz, 2H, H-6), 3.73 (qd, J = 7.1, 1.8 Hz, 6H, H-9), 2.56 (s, 3H, H-1), 2.06 (p, J = 7.3 Hz, 2H, H-7), 1.12 (td, J = 7.0, 1.9 Hz, 9H, H-10), 0.59–0.51 (m, 2H) ppm (see Figure 3 for proposed numbering assignment).

#### 2.2.4. Synthesis of MSN-[Chol][Cl], MSN-[1-MiM][Cl] and MSN-[3-Pic][Cl]

These materials were prepared by the following general procedure: calcined MSN-OH (0.50 g) was heated at 150 °C under reduced pressure for 2 h to remove the physiosorbed water. After cooling down to room temperature, an excess of the ionic liquid derivative (0.40 g) in toluene (8 mL), or in a mixture of ethanol and acetonitrile in the case of Si-[Chol][Cl] (due to its solubility), was added and the mixture was stirred under microwave irradiation at 100 °C for 1 h. The solid was separated by centrifugation (5000 rpm, 15 min) and washed with ethanol four times. The resultant material was dried overnight at 80 °C.

MSN-[Chol][Cl]: The data characterization of this material is in accordance with that previously described [66].

MSN-[1-MiM][Cl]: Analysis found (%): C, 10.89; N, 3.20; H, 2.83;

^1^H NMR (400 MHz, D_2_O + NaOH, rt) δ = 7.19 (d, J = 2.0 Hz, 1H, H-4), 7.12 (d, J = 2.0 Hz, 1H, H-3), 3.87 (d, 2H, H-5), 3.60 (s, 3H, H-1), 1.69–1.60 (m, 2H, H-6), 0.12–0.05 (m, 2H, H-7) ppm (see Figure 2 for numbering assignment).

FTIR (KBr/cm^−1^): 3367 (br), 3155 (sh), 3106 (sh), 2894 (sh), 1635 (s), 1575 (s), 1456 (m), 1049 (vs), 927 (m), 788 (m) 709 (w), 643 (w), 612 (s), 555 (m).

MSN-[3-Pic][Cl]): Anal. Found (%): C, 19.75; N, 2.39; H, 2.39;

^1^H NMR (400 MHz, D_2_O + NaOH, rt) δ = 8.57 (s, 1H, H-2), 8.52 (d, J = 6.1 Hz, 1H, H-5), 8.22 (d, J = 8.0 Hz, 1H, H-3), 7.79 (dd, J = 8.1, 6.0 Hz, 1H, H-4), 4.40 (t, J = 7.2 Hz, 2H, H-6), 2.42 (s, 3H, H-1), 2.00–1.90 (m, 2H, H-7), 0.33–0.26 (m, 2H, H-8) ppm (see Figure 3 for numbering assignment).

FTIR (KBr/cm^−1^): 3419 (br), 2940 (sh), 1638 (s), 1507 (s), 1487 (m), 1447 (w), 1399 (m), 1073 (vs), 929 (w), 803 (s), 734 (w), 686 (s), 572 (w), 456 (vs).

#### 2.2.5. Synthesis of the Sodium Salt of Ciprofloxacin ([Na][Cip])

In a round-bottom flask, 0.50 g of ciprofloxacin (1.51 mmol) was stirred in 250 mL of dry ethanol. After partial dissolution of the antibiotics, a 25 mL aqueous solution of equimolar amount of sodium hydroxide (60.4 mg) was added dropwise. The mixture was stirred for 24 h, at room temperature, and the solvent removed under reduced pressure. The resulting powder was suspended in ethanol, filtered, evaporated under reduced pressure and dried overnight under vacuum to afford a white solid as the sodium salt of ciprofloxacin ([Na][Cip]).

#### 2.2.6. Synthesis of MSN-[Chol][Cip], MSN-[1-MiM][Cip] and MSN-[3-Pic][Cip] with Ciprofloxacin (Cip)

These materials were prepared by anionic exchange following the method described previously [66] by reaction of the materials MSN-[Chol][Cl], MSN-[1-MiM][Cl] and MSN-[3-Pic][Cl] with an aqueous solution of the sodium salt of ciprofloxacin ([Na][Cip]).

MSN-[Chol][Cip]: The data characterization of this material is in accordance with the previously reported [66].

MSN-[1-MiM][Cip]: Analysis found (%): C, 24.41; N, 5.65; H, 3.25;

^1^H NMR (400 MHz, D_2_O + NaOH, rt) δ = 8.11 (s, H-h), 7.51 (d, H-c), 7.25 (d, H-d), 7.07 (s, H-4), 7.01 (s, H-3), 3.75 (m, H-5), 3.48 (s, H-1), 3.26 (s, H-e), 2.86 (s, H-b), 2.64 (s, H-a), 1.60–1.48 (m, H-6), 0.95 (t, H-f), 0.74 (t, H-g) ppm (see Figure 2 for numbering assignment).

FTIR (KBr/cm^−1^): 3422 (br), 3155 (sh), 3045 (sh), 3016 (sh), 1619 (s), 1590 (s), 1543 (m), 1500 (m), 1380 (m), 1333 (w), 1090 (vs), 868 (m), 785 (m) 723 (m), 651 (w) 622 (m), 457 (vs).

MSN-[3-Pic][Cip]: Analysis found (%): C, 27.81; N, 4.87; H, 3.01;

^1^H NMR (400 MHz, D_2_O + NaOH, rt) δ = 8.32–8.22 (m, H-2, H-5), 8.11 (s, H-h), 7.94 (d, H-3), 7.50 (d, H-c, H-4), 7.25 (d, H-d), 4.13 (t, H-6), 3.26 (m, H-e), 2.86 (t, J = 4.5 Hz, H-b), 2.64 (t, J = 4.8 Hz, H-a), 2.14 (s, H-1), 1.68 (t, H-7), 0.96 (d, H-f), 0.74 (s, H-g) ppm (see Figure 3 for numbering assignment).

FTIR (KBr/cm^−1^): 3425 (br), 3045 (sh), 3018 (m), 2961 (sh), 2850 (sh), 1620 (s), 1590 (s), 1543 (m), 1500 (s), 1474 (m), 1378 (s), 1078 (vs), 938 (w), 785 (s), 723 (s), 683 (w), 566 (m), 459 (vs).

### 2.3. Antimicrobial Studies

The antimicrobial activity of the materials was tested against strains of sensitive and resistant *Klebsiella pneumoniae* (HS31 and HS16, respectively) as well as sensitive and resistant strains of *Enterococcus* spp. (FC89-8R e FC89-10R, respectively). *K pneumoniae* samples come from nosocomial infections, while *Enterococcus* spp. originate from infections of free-range chickens. The antimicrobial susceptibilities for all isolates were determined using the Kirby–Bauer disk diffusion method in accordance with EUCAST standards (2022) [68]. Briefly, the bacteria were seeded on BHI agar and incubated at 37 °C for 24 h to promote their growth. Then, using a swab, 1 to 2 colonies were picked and diluted in 3 mL of sterile saline solution (0.9% NaCl) until reaching a concentration equivalent to 0.5 on the McFarland scale. Subsequently, a homogeneous smear was made on an MH agar plate (Mueller-Hinton agar). Finally, antibiotic disks were properly spaced on the plate using forceps. The plates were then stored in an incubator at 37 °C for 24 h. After this time, an inhibition halo was observed around each disk, resulting from the effect that the antibiotic had on the bacteria under study. The diameters (mm) of the halos were measured in order to compare the results with regulatory standards and determine the resistance exhibited by the bacteria to each antibiotic.

A total of 17 antibiotics were tested for *K. pneumoniae*: ceftazidime (CAZ) (10 μg), cefepime (FEP) (30 μg), ertapenem (ERT) (10 μg), amikacin (AK) (30 μg), ciprofloxacin (CIP) (5 μg), trimethoprim–sulfamethoxazole (SXT) (1.25/23.75 μg), tetracycline (TE) (30 μg), cefoxitin (FOX) (30 μg), gentamicin (CN) (10 μg), imipenem (IMP) (10 μg), meropenem (MEM) (10 μg), aztreonam (ATM) (30 μg), amoxicillin-clavulanic acid (AUG) (20–10 μg), chloramphenicol (CHL) (30 μg), ampicillin (AMP) (10 μg), cefotaxime (CTX) (5 μg) and nalidixic acid (NA) (30 μg). The phenotypical profile of *K. pneumoniae* strains is shown in Table S1.

A total of 13 antibiotics were tested for *Enterococcus* spp.: tetracycline (TET) (30 μg), vancomycin (VA) (30 μg), erythromycin (ERY) (15 μg), gentamicin (CN) (120 μg), chloramphenicol (CHL) (30 μg), ciprofloxacin (CIP) (5 μg), quinupristin-dalfopristin (QD) (15 μg), teicoplanin (TEI) (30 μg), ampicillin (AMP) (10 μg), nitrofurantoin (F) (300 μg), rifampicin (RD) (5 μg), phosphomycin (FOS) (200 μg) and linezolid (LNZ) (30 μg). The phenotypical profile of *Enterococcus* spp. strains is shown in Table S2.

We utilized the microdilution technique to ascertain the minimum inhibitory concentration (MIC) of ciprofloxacin. Initially, each strain was introduced onto Brain Heart Infusion agar and allowed to incubate at 37 °C for 24 h. Subsequently, the bacterial cells were subcultured in tubes containing Mueller–Hinton broth and subjected to overnight incubation at 37 °C with 150 rpm using the ES-20/60 Orbital Shaker-Incubator from Biosan in Riga, Latvia. The overnight culture was then diluted in fresh MH broth to attain a turbidity standard of 0.5 McFarland, employing a spectrophotometer. Next, ciprofloxacin was diluted in sterilized distilled water, generating various concentrations ranging from 0 µg/mL to 8182 µg/mL. To assess bacterial growth, 75 µL of each ciprofloxacin concentration was dispensed into a polystyrene flat-bottom 96-well plate. Simultaneously, 75 µL of the bacterial suspension was placed in the same 96-well microtiter plate. The plates were subsequently incubated for 24 h at 37 °C with 150 rpm. Bacterial growth was quantified at 490 nm using a microplate reader, specifically the BioTek ELx808U from BioTek in Winooski, VT, USA. The MIC was determined as the lowest concentration of ciprofloxacin that effectively impeded bacterial growth. Triplicates were performed for every different concentration tested on each nanomaterial.

### 2.4. Cytotoxicity Studies

#### 2.4.1. Cell Culture

Normal dermal human fibroblasts were acquired from ATCC (Manassas, VA, USA) and cells were cultured in Dulbecco’s modified Eagle medium (DMEM, Thermo Fisher Scientific). The supplemented media are referred to from this point on as DMEM for simplicity. All experiments undertaken at 37 °C, 5% (*v*/*v*) CO_2_ and 99% (*v*/*v*) humidity in the dark.

#### 2.4.2. Antiproliferative Studies

Cells were seeded in a 96-well plate at a density of 7500 cells/well, and after 24 h were submitted to a concentration range between 0.1–50 µM of MSN-API-OSILs. After 48 h incubation, cellular viability was inferred with the Cell Titer 96^®^ Aqueous One solution cell proliferation assay (Promega, Madison, WI, USA) according to the manufacturer’s instructions and procedures previously described [69]. The absorbance at 490 nm of the produced Formazan was quantified with a Tecan microplate reader, Infinite M200 (Tecan, Mannedorf, Switzerland), and the analysis of the dose–response curves to determine the relative IC_50_ was performed with GraphPad Prism 8 software (GraphPad Software, La Jolla, CA, USA). The cytotoxicity studies were conducted in triplicate for all compounds, and each concentration tested in duplicate.

## 3. Results and Discussion

### 3.1. Synthesis and Characterization

**Scheme 1:**
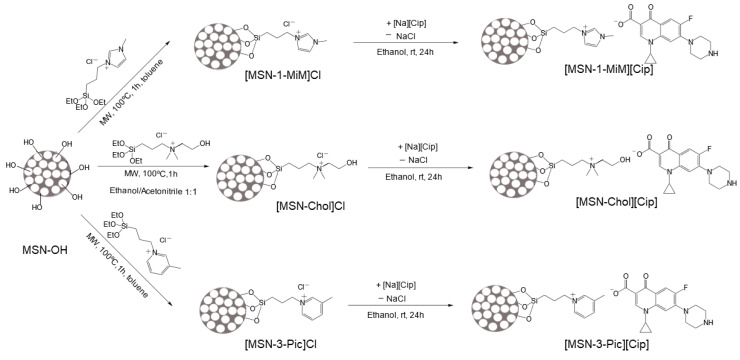
General synthetic scheme for the preparation of the nanomaterials functionalized with anionic ciprofloxacin.

Our group has recently reported the functionalization of pristine mesoporous silica nanoparticles (MSNs) with a covalently linked choline derivative cation and ciprofloxacin as anion [66]. In this work, the effect of the cation functionalization, such as choline (for comparison purposes), methylimidazolium and picolinium derivatives, on the antimicrobial activity of ciprofloxacin (Cip) is evaluated. More sustainable synthetic methodology based on microwave heating for the preparation of the cation’s precursors as well as their functionalization on MSNs enabled faster reactions, higher yields and less solvent required. Three final hybrid nanomaterials containing the three different cations and Cip as anion, designated as [MSN-Chol][Cip], [MSN-1-MiM][Cip] and [MSN-3-Pic][Cip], were prepared according to the strategic approach illustrated in Scheme 1.

All nanomaterials were characterized by FTIR, solution ^1^H NMR, elemental analysis, XRD and N_2_ adsorption-desorption isotherms at 77 K. The data characterization obtained for [MSN-Chol][Cip] and its precursor [MSN-Chol][Cl] is consistent with that previously described [66].

The FTIR spectra obtained for [MSN-1-MiM][Cip] and its precursors are shown in Figure S1. The typical vibrational patterns for the silica network in pristine MSNs can be identified at 1087, 962, 798 and 460 cm^−1^, corresponding to ν_as_(Si–O–Si), ν_as_(Si–OH), ν_s_(Si–O–Si) and δ(Si–O–Si), respectively. The spectrum of [MSN-1-MiM][Cl] contains additional peaks in the range of 3400–2900 cm^−1^, which can be attributed to ν(C-H) as well as the ν(N-H), and at 1575 and 1456 cm^−1^, indicative of the C-H vibrational modes of the imidazolium moiety. New peaks in the region between 710 and 550 cm^−1^ can be ascribed to the stretching of ν(N-H) and aliphatic ν(C-H) vibrations. In the [MSN-1-MiM][Cip] spectrum, new peaks can be observed in the aromatic region, which is indicative of the correct functionalization of the material with the antibiotic. Furthermore, the peak at 1543 cm^−1^ can be assigned to ν_as_(COO^−^) of ciprofloxacin.

Figure S2 illustrates the FTIR spectra of [MSN-3-Pic][Cip] and its precursors. The spectrum of [MSN-3-Pic]Cl shows the appearance of notorious and particularly sharp peaks at 1507, 1487 and 686, cm^−1^, which can be attributed to the ν(C-C) stretches of the aromatic ring, the in-plane δ(C-H) bending and the out-of-plane δ(C-H) bending. The alkyl ν(C-H) vibrations can also be identified around the 2900 cm^−1^ region. As for the material subsequently functionalized with ciprofloxacin, it is possible to note an increase in the aromatic region, as well as the appearance of the very characteristic peak at 1543 cm^−1^ ascribed to the ν_as_(COO^−^) of anionic ciprofloxacin.

**Figure 4:**
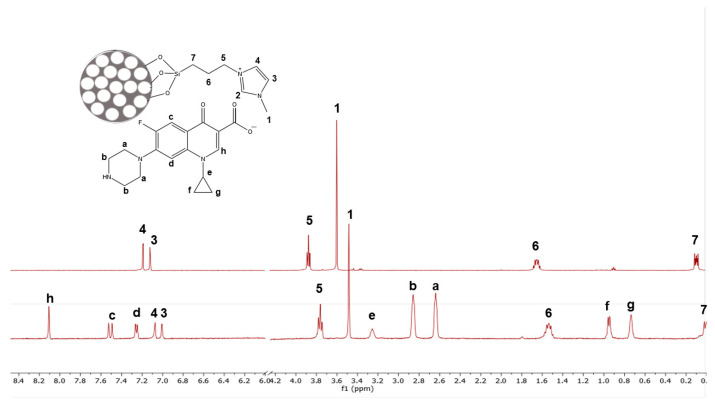
^1^H NMR spectra of [MSN-1-MiM]Cl (top) and [MSN-1-MiM][Cip] (bottom).

**Figure 5:**
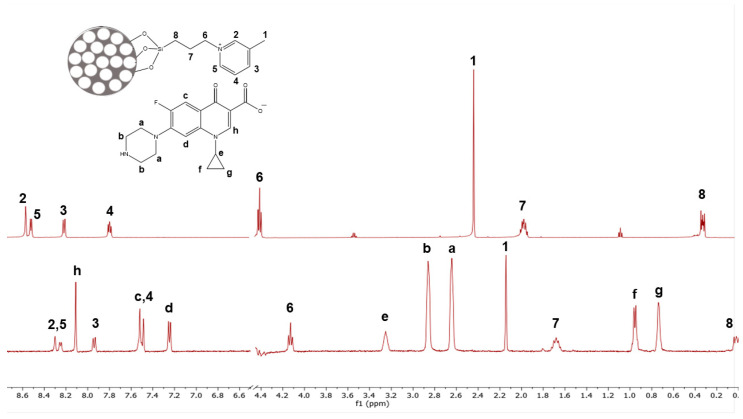
^1^H NMR spectra of [MSN-3-pic]Cl (top) and [MSN-3-pic][Cip] (bottom).

The ^1^H NMR solution spectra obtained (following the method described by Crucho et al. [70]) for [MSN-1-MiM]Cl/[MSN-1-MiM][Cip] and [MSN-3-pic]Cl/[MSN-3-pic][Cip] are shown in Figure 4 and Figure 5, respectively. The spectra of [MSN-1-MiM]Cl and [MSN-3-pic]Cl contain all the peaks corresponding to the respective functionalized ionic liquid moiety. The exchange of chloride by Cip anions is confirmed by the presence of the proton’s peaks assigned to the antibiotic in both [MSN-1-MiM][Cip] and [MSN-3-pic][Cip] spectra.

The loading of functionalized MSN materials was determined through elemental analysis, and later the results were compared with the NMR information. The values of the loading obtained through elemental analysis are summarized in Table 1. For [MSN-Chol]Cl, the C/N molar ratio obtained was 8.6 (the theoretical value for all ethoxy groups bounded to the surface is 7), which suggests that the anchorage occurs mainly through two ethoxy groups; the content of choline derivatives moiety was 1.0 mmol g^−1^ based on nitrogen value, a higher content than the one obtained for the analogous material described previously (0.73 mmol g^−1^ [66]). For materials [MSN-1-MiM]Cl and [MSN-3-Pic]Cl the C/N ratios were 3.9 (theoretical = 3.5) and 9.6 (theoretical = 8), respectively, indicating that the picolinium cation derivative is bounded to the surface also mainly through two ethoxy groups. In relation to the organic cations content, a higher value for the picolinium derivative (1.7 mmol g^−1^) was obtained in comparison with the choline (1.0 mmol g^−1^) and imidazolium derivatives (1.1 mmol g^−1^). Considering these loadings of organic cations, the C/N molar ratios and N content in the final hybrid materials, it is possible to estimate the content of Cip as counter-ion in the following materials: [MSN-Chol][Cip] (0.60 mmol g^−1^), [MSN-1-MiM][Cip] (0.66 mmol g^−1^) and [MSN-3-Pic][Cip] (1.34 mmol g^−1^) (Table 1). These results mean that around 79% of picolinium cation derivatives contain Cip as counter-ion, while for choline and imidazolium cations this value is around 60%. The loadings of anchored guests determined through NMR spectroscopy were lower than those obtained through elemental analysis: 0.78 mmol g^−1^ for [MSN-Chol]Cl, 0.92 mmol g^−1^ for [MSN-1-MiM]Cl and 1.51 mmol g^−1^ for [MSN-3-Pic]Cl. This difference can be justified by experimental error of the procedure. However, in the final materials, comparing the ratio between the integration of the ciprofloxacin moiety protons and cation protons grafted on the surface, it is possible to confirm that the correlation approaches the same proportion already denoted through elemental analysis.

The N_2_ adsorption–desorption isotherms of nanomaterials and corresponding pore size distributions (Figure S3) present features as in previous work, namely those of the pristine MSN are typical of mesoporous nanomaterials with reasonably uniform mesopore diameter inside small nanoparticles. The results of the analysis of the N_2_ adsorption/desorption isotherms by the BET method, using criteria recommended by IUPAC [71] and by NLDFT using the Quantachrome software ASiQwin (Table 2), are in agreement with the higher organic loading obtained for [MSN-3-Pic]Cl, since this material revealed more pronounced changes in the textural properties than [MSN-Chol]Cl and [MSN-1-MiM]Cl. In the case of [MSN-Chol]Cl, the changes are more marked than in our previous work [66] where the final content of the choline derivative cation was lower. In all cases, there is an additional decrease of A_BET_ with the ionic exchange compared with their precursors.

The XRD diffraction patterns for the precursor and for the functionalized nanomaterials are shown in Figure S4, and are typical of mesoporous silica nanoparticles, with the main peak broader and less intense than those obtained for bigger particles and with comparable average pore diameters, reflecting the usual lesser ordering of the mesopores inside the small nanoparticles.

### 3.2. Antimicrobial and Cytotoxicity Studies

**Figure 6:**
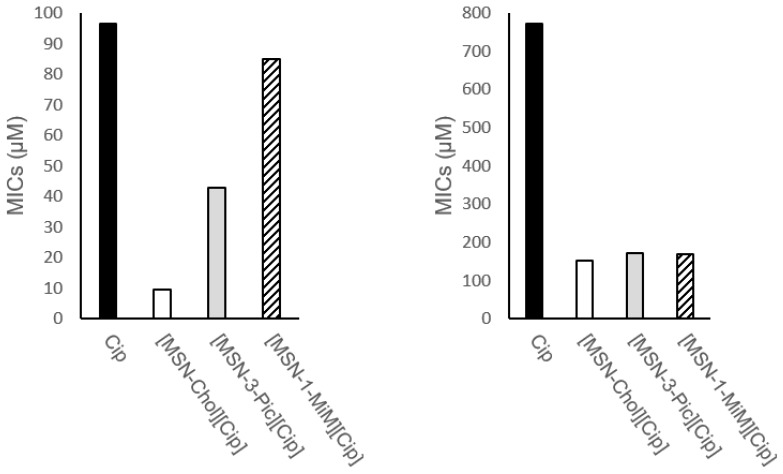
Minimum inhibitory concentration obtained against sensitive strains of *K. pneumoniae* (**left**) and resistant strains of *K. pneumoniae* (**right**).

**Figure 7:**
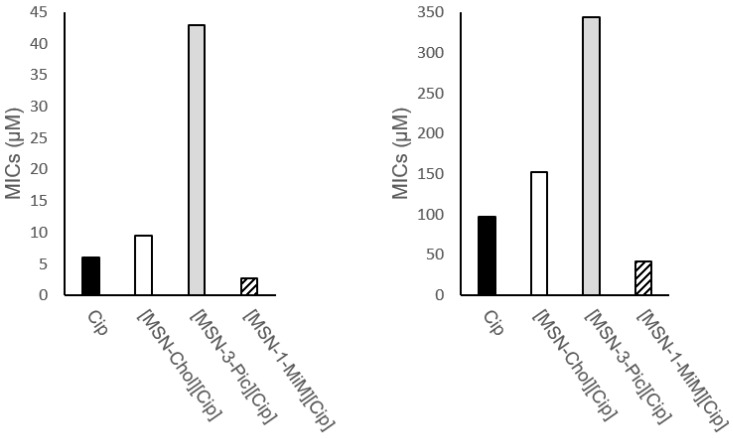
Minimum inhibitory concentration obtained against sensitive strains of *Enterococcus* spp. (**left**) and resistant strains of *Enterococcus* spp. (**right**).

The minimum inhibitory concentration of the nanomaterials was determined against Gram-positive *Enterococcus* spp. and Gram-negative *Klebsiella pneumoniae*. To determine the lowest concentration that inhibits bacterial growth, a range of solutions of different concentrations of the nanomaterials were incubated with the bacterial suspensions. The assays started at the lowest concentration of the nanomaterials, following each subsequent assay with a solution twice as concentrated as the one used in the previous assay. Therefore, the MIC was determined as the lowest concentration found for each active nanomaterial that effectively impeded bacterial growth. Upon finding such concentration, the loading of anchored guests and anionic ciprofloxacin in each individual set of nanomaterials (Table 1) is taken into account. As such, the value for the MIC is normalized regarding the amount of anionic ciprofloxacin estimated in each respective set of nanomaterials. The results are presented in Figure 6 and Figure 7, with all data being compared to ciprofloxacin, used as positive control.

In the case of *K. pneumoniae*, all nanomaterials containing anionic ciprofloxacin outperformed free ciprofloxacin. Regarding the action against the sensitive *K. pneumoniae* strains, [MSN-1-MiM][Cip] demonstrated only a slight improvement in inhibiting the bacterial growth in comparison with ciprofloxacin. However, [MSN-3-Pic][Cip] illustrates a reduction to more than half in the concentration needed to inhibit bacterial growth, while [MSN-Chol][Cip] showed a tenfold decrease in the concentration needed for inhibition. Regarding the resistant *K. pneumoniae* strain, it is possible to note an increase for ciprofloxacin required to inhibit the bacterial growth from 96.5 µM to 772.6 µM. In the case of nanomaterials functionalized with anionic ciprofloxacin, it is observed that it outperformed compared to free ciprofloxacin against resistant *K. pneumoniae*. Similar inhibition patterns were identified in every set of nanomaterials, as a five-fold decrease in concentration was found for all materials.

The nanomaterials demonstrated a lower potency against Gram-positive *Enterococcus* spp., regardless of the sensitivity of the strain. Against sensitive *Enterococcus* spp., both [MSN-Chol][Cip] and [MSN-3-Pic][Cip] are less efficient compared to free ciprofloxacin. However, the [MSN-1-MiM][Cip] demonstrated a slight improvement over ciprofloxacin, with a three-fold reduction on the MIC value. A similar inhibition pattern was observed in all the materials against sensitive strains of *Enterococcus* spp. In this set of tests, both [MSN-Chol][Cip] and [MSN-3-Pic][Cip] were not able to outperform free ciprofloxacin; however, [MSN-1-MiM][Cip] showed an improvement by reducing the concentration needed for inhibition by half. This result appears to suggest that, against Gram-positive bacteria, the presence of the 1-methylimidazolium cation plays a role in activating or enhancing the ciprofloxacin.

Pristine MSNs and the precursors of the final materials—[MSN-Chol]Cl, [MSN-3-Pic]Cl and [MSN-1-MiM]Cl—were also tested against sensitive and resistant strains of *K. pneumoniae* and *Enterococcus* spp. and no noticeable effect was detected on the bacterial growth. On the upper limit, concentrations of up to 1 mg/mL were tested and no inhibition on bacterial growth was observed, which is consistent with the fact that only the addition of the antibiotic to the final material showed antibacterial activities.

The values of the minimum inhibitory concentrations (MIC, in µM) for the most active compounds are displayed in Table 3.

The results point towards a correlation between the presence of the nanoparticle and the increase in the potency of the antibiotic, even if the improvement in results could not be fully extended to the tests against Gram-positive bacteria. This latter result can be somewhat correlated to the larger and more complex bacterial wall present in Gram-positive bacteria, which might hinder the permeability of the antibiotic as well as making it more difficult for nanoparticle system to interact with the microorganism. However, due to the nature of the antibacterial studies performed, we are not able to make a thorough assessment on the overall effect of the different organic cations grafted on the surface. Simultaneously, because the MICs were calculated by doubling the concentration of the active materials in every subsequent assay, more detailed studies are needed to reach the most precise measurement of the MICs of each compound. Nonetheless, because in this study we could not determine the lowest possible concentration of inhibition, we postulate that more detailed studies—spawning shorter ranges of concentrations—should only improve the results, revealing an even lower concentration needed to hamper the bacterial growth.

Cytotoxicity assays were performed using the three prepared mesoporous silica nanoparticles loaded with the antibiotic, as well as the precursor materials and free ciprofloxacin. The selection of human primary dermal fibroblasts is considered a good surrogate model test system for human healthy cells. In general, none of the compounds displayed any in vitro cytotoxicity at high concentrations (50 μM). The low cytotoxicity at such high concentration demonstrates that this approach seems to be a good nanoplatform for future pharmaceutical applications.

## 4. Conclusions

In this work, a protocol for the synthesis of ionic liquids using microwave-assisted heating was employed. In comparison with conventional organic synthesis procedures—which typically require several hours at reflux temperatures—the present work successfully provides a greener alternative, with reliable results, satisfactory yields, faster reactions and easier purifications. The obtained ionic liquids were grafted on the surface of mesoporous silica nanoparticles to functionalize the surface with organic cations, combining with ciprofloxacin as anion. The materials were extensively characterized through various techniques, including FTIR, solution ^1^H NMR, elemental analysis, XRD and N_2_ adsorption at 77 K to prove the desired structures as well as important parameters.

All prepared compounds are non-toxic according to the results of in vitro cytotoxicity using human fibroblasts.

The antimicrobial activities were determined against Gram-negative *K. pneumoniae* and Gram-positive *Enterococcus* spp. both with resistant and sensitive strains. Against the Gram-negative bacteria, all sets of nanomaterials were able to outperform free ciprofloxacin, with significant reduction in the minimum inhibitory concentration. The material [MSN-Chol][Cip] showed a tenfold increase in the antibacterial activity against sensitive *K. pneumoniae*, compared to free ciprofloxacin. For the case of resistant Gram-negative strains of *K. pneumonia*, all sets of nanomaterials showed a five-fold decrease in the concentration needed to hamper bacterial growth. Against resistant and sensitive *Enterococcus* spp. strains, only the material [MSN-1-MiM][Cip] was able to demonstrate a slight improvement over free ciprofloxacin. Further studies should be conducted to evaluate the MICs with more precision, as well as any possible correlation between the choice of IL/antibiotic, which might allow the tuning of specific properties assisting the development of more potent formulations and directed therapies.

## Figures and Tables

**Table 1:** Values for the loading of organic content of the functionalized nanomaterials obtained by elemental analysis. In the case of the materials functionalized with ciprofloxacin, the value refers to the amount of ciprofloxacin expected to be present in the material.

Material	Loading (mmol g^−1^)
[MSN-Chol]Cl	1.02
[MSN-Chol][Cip]	0.60
[MSN-1-MiM]Cl	1.13
[MSN-1-MiM][Cip]	0.66
[MSN-3-Pic]Cl	1.77
[MSN-3-Pic][Cip]	1.34

**Table 2:** Values of A_BET_, D_p_ and V_p_ for the nanomaterials ^1^.

Material	A_BET_ (m^2^g^−1^)	D_p_ (nm)	V_p_ (cm^3^g^−1^)
Pristine MSN (Chol)	751	3.3	0.40
Pristine MSN (1-MiM and 3-Pic)	721	3.3	0.42
[MSN-Chol]Cl	280	2.5	0.08
[MSN-Chol][Cip]	57	-	-
[MSN-1-MiM]Cl	203	3.4	0.08
[MSN-1-MiM][Cip]	40	-	-
[MSN-3-Pic]Cl	48	-	-
[MSN-3-Pic][Cip]	39	-	-

^1.^ A_BET_—specific surface area obtained by the BET method; D_p_ and V_p_—pore diameter and cumulative pore volume of pores up to 8 nm obtained from the NLDFT pore size distributions.

**Table 3:** Values of the minimum inhibitory concentration for the most active compounds. Values are represented in units of µM.

Material	Sensitive *K. pneumoniae*	Resistant *K. pneumoniae*	Sensitive *Enterococcus* spp.	Resistant *Enterococcus* spp.
Ciprofloxacin (control)	96.5	772.6	6.0	96.5
[MSN-Chol][Cip]	9.5	152.8	9.5	152.8
[MSN-3-Pic][Cip]	42.9	171.7	42.9	343.4
[MSN-1-MiM][Cip]	84.5	169.0	2.6	42.2

## Data Availability

Data are available in a publicly accessible repository.

## Supplementary Materials

The following supporting information can be downloaded at: https://www.mdpi.com/article/10.3390/pharmaceutics15071934/s1, Figure S1: FTIR spectra of (A) pristine MSNs, (B) [MSN-1-MiM]Cl, and (C) [MSN-1-MiM][Cip]; Figure S2: FTIR spectra of (A) pristine MSNs, (D) [MSN-3-Pic]Cl and (E) [MSN-3-Pic][Cip]; Figure S3: Nitrogen adsorption–desorption isotherms determined at 77 K on the prepared nanomaterials: (left) pristine MSN (Chol) and derived materials, (right) pristine MSN (1-MiM and 3-Pic) and derived materials. NLDFT pore size distributions are inserted into the figures; Figure S4: X-ray diffraction patterns of the prepared nanomaterials: (left) pristine MSN (Chol) and derived materials; (right) pristine MSN (1-MiM and 3-Pic) and derived materials. Table S1. Phenotypical profile of *K. pneumoniae* strains of bacteria and their respective resistance profile obtained. S—means sensitivity to the antibiotic; R—means resistance to the antibiotic; Table S2. Phenotypical profile of *Enterococcus* spp. strains of bacteria and their respective resistance profile. S—means sensitivity to the antibiotic; R—means resistance to the antibiotic.
