# Supplementary material for: In Vitro Antimicrobial Studies of Mesoporous Silica Nanoparticles Comprising Anionic Ciprofloxacin Ionic Liquids and Organic Salts
Source: Pharmaceutics. 2023 Jul 12;15(7):1934. doi: 10.3390/pharmaceutics15071934 (PMC10385687; doi:10.3390/pharmaceutics15071934)
Supplement: Supplementary file 1 [file pharmaceutics-15-01934-s001.zip › pharmaceutics-2454194-supplementary.pdf]

pharmaceutics

Supplementary Materials: In Vitro Antimicrobial Studies of Mesoporous Silica Nanoparticles Comprising Anionic Ciprofloxacin Ionic Liquids and Organic Salts

Luís Filipe, Telma de Sousa, Dário Silva, Miguel M. Santos, Manuela Ribeiro Carrott, Patrícia Poeta, Luís C. Branco and Sandra Gago

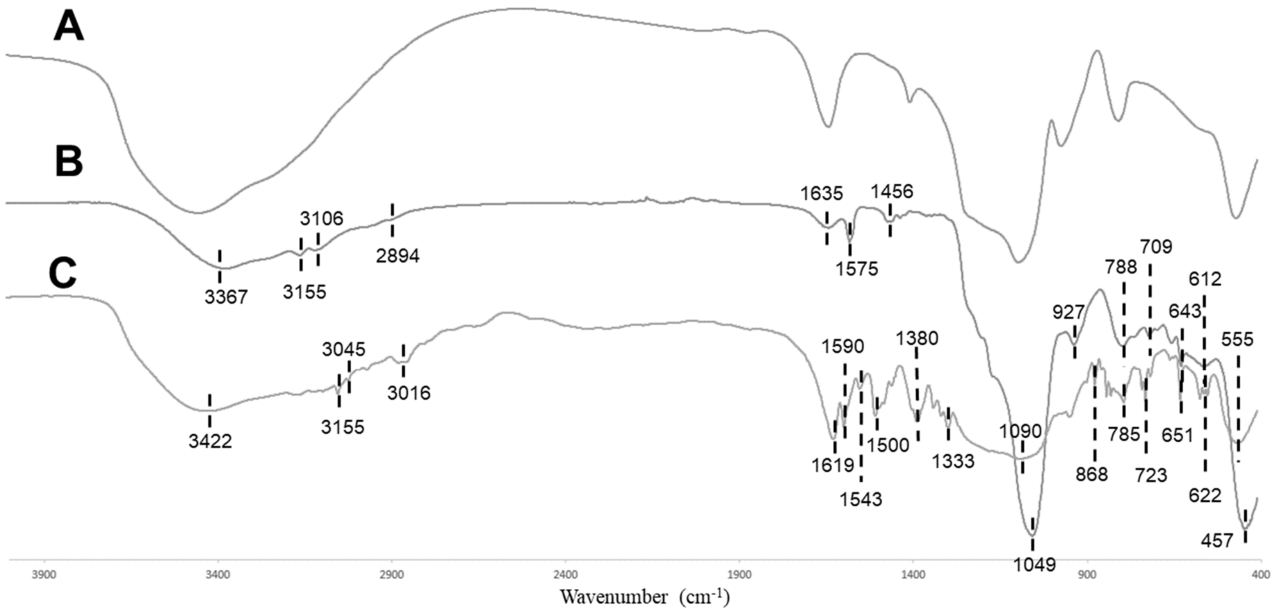

Figure S1. FTIR spectra of (A) pristine MSNs; (B) [MSN-1-MiM]Cl; and (C) [MSN-1-MiM][Cip].

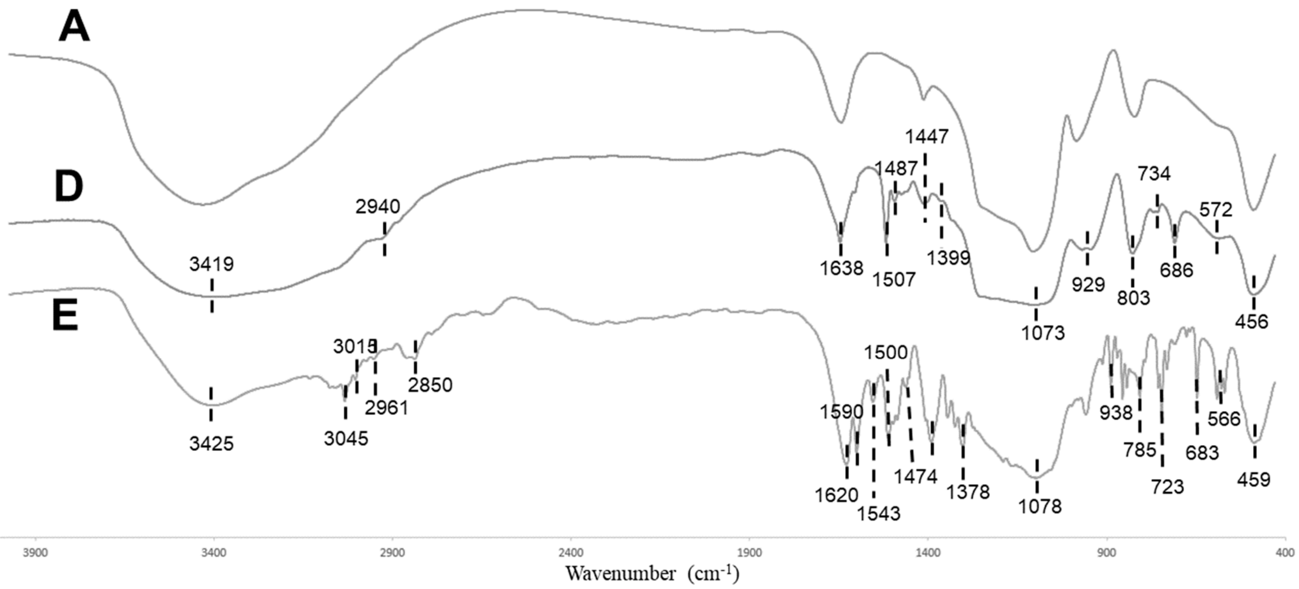

Figure S2. FTIR spectra of (A) pristine MSNs; (D) [MSN-3-Pic]Cl; and (E) [MSN-3-Pic][Cip].

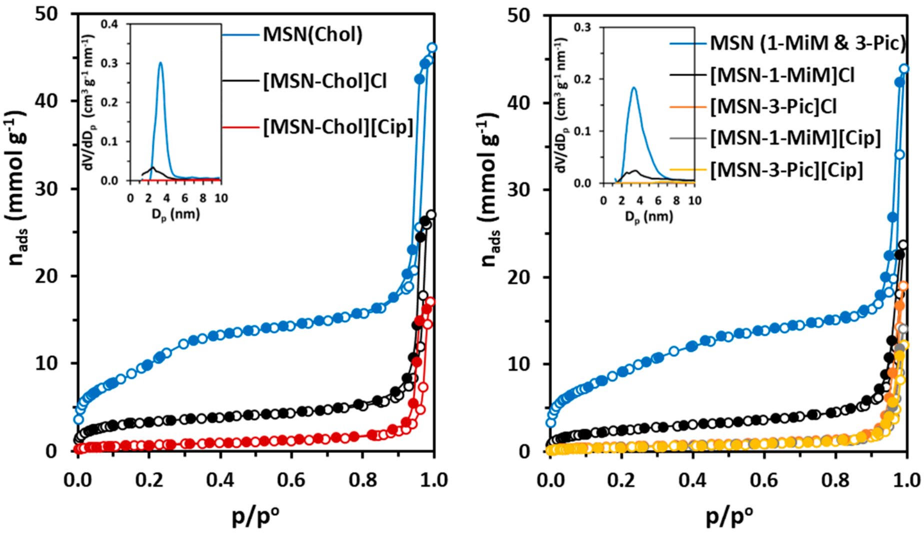

Figure S3. Nitrogen adsorption-desorption isotherms determined at 77 K on the prepared nanomaterials: (left) pristine MSN (Chol) and derived materials; (right) pristine MSN (1-MiM and 3-Pic) and derived materials. NLDFT pore size distributions are inserted into the figures.

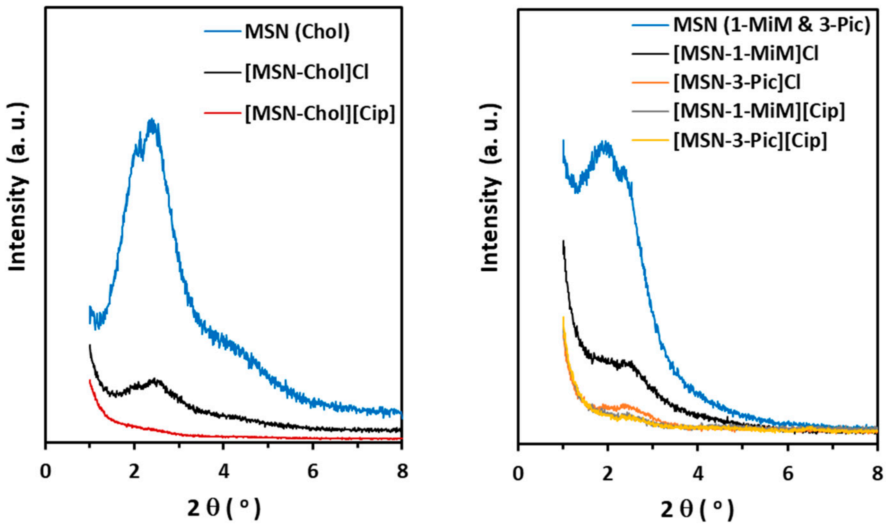

Figure S4. X-ray diffraction patterns of the prepared nanomaterials: (left) pristine MSN (Chol) and derived materials; (right) pristine MSN (1-MiM and 3-Pic) and derived materials.

Table S1. Phenotypical profile of *K. pneumoniae* strains of bacteria and their respective resistance profile obtained. S – means sensitivity to the antibiotic; R – means resistance to the antibiotic;

Antibiotic / ***Strain***	*AMP*	*AUG*	*FOX*	*CAZ*	*CTX*	*FEP*	*ATM*	*MEM*	*ERT*	*IMP*	*AK*	*CN*	*TET*	*CIP*	*NA*	*SXT*	*CHL*
HS16	R	R	S	R	R	R	R	S	S	S	S	R	S	R	R	R	R
HS31	R	S	S	S	S	S	S	S	R	S	S	S	S	S	S	S	S

Table S2. Phenotypical profile of *Enterococcus* spp. strains of bacteria and their respective resistance profile. S – means sensitivity to the antibiotic; R – means resistance to the antibiotic;

Antibiotic / ***Strain***	*AMP*	*VAN*	*TEC*	*E*	*TET*	*CIP*	*F*	*RD*	*FOS*	*C*	*QD*	*LNZ*	*CN*
FC89-8R	*S*	*S*	*S*	*S*	*R*	*S*	*S*	*S*	*S*	*S*	*S*	*S*	*S*
FC89-10R	*S*	*S*	*S*	*R*	*R*	*R*	*S*	*R*	*S*	*S*	*S*	*S*	*S*
